# Causes and preventive measures for fire-related injuries in intensive care units: a systematic review

**DOI:** 10.7189/jogh.15.04043

**Published:** 2025-02-28

**Authors:** JiaFang Wu, YuQing Yao, XiuLing Shang

**Affiliations:** 1 Department of Critical Care Medicine, Shengli Clinical Medical College of Fujian Medical University, Fuzhou University Affiliated Provincial Hospital, Fuzhou, China; 2 Shengli Clinical Medical College of Fujian Medical University, Fuzhou, China

## Abstract

**Background:**

Intensive care unit (ICU) fires are a significant public health concern which often receives insufficient attention. However, they notoriously challenging to manage, with a high risk of casualties and substantial economic damage. Despite their severity, research on ICU fires remains scarce.

**Methods:**

In this systematic review, we queried PubMed, Cochrane, Embase, Web of Science, and Web of Baidu Academic for studies on ICU or hospital fires published from the inception of each database to 5 July 2024. We then narratively summarised the causes of ICU fires and the details of ICU fire prevention and evacuation.

**Results:**

We examined 2602 articles, of which 30 met our inclusion criteria. We summarised the causes and injuries of 18 ICU fires. The most common causes were electrical faults and oxygen cylinder faults, while the prevention and response strategies for ICU fires included regular maintenance of electrical and oxygen equipment, installation of automatic fire fighting systems, reasonable arrangement of patient beds, vertical and horizontal evacuation route planning, online and offline fire safety training, and intelligent evacuation simulation.

**Conclusions:**

We found that the primary causes of fires in ICUs are electrical malfunctions and oxygen-related incidents. It is essential to implement preventive measures such as regular inspection of electrical equipment, proper maintenance of wiring, and ensuring all oxygen systems are handled according to strict safety protocols. Additionally, ensuring that all ICU staff are adequately trained in emergency response protocols can significantly improve patient outcomes in the event of a fire.

Hospital fires remain a major issue in countries worldwide, as they can negatively impact economies and societies with enormous costs to people and the environment. According to the US National Fire Protection Association, fire departments respond to an average of 5750 structure fires in health care facilities every year [1,2]. These incidents cause an annual average of two deaths and 157 injuries [1,2]. Since the outbreak of the COVID-19 pandemic in March 2020, they have caused the deaths of over 200 people in countries worldwide, most of whom were patients extremely ill with the novel coronavirus [3–5]. The incidence of hospital fire outbreaks during the pandemic has increased approximately 2-fold compared with the last ten years [5–8]. Unfortunately, there are currently no globally consistent methods of reporting, collecting, and analysing losses (economic, human, or otherwise) due to fires [9]. Such limited or inconsistent reporting may have led to underestimations of the prevalence of such events. Among all hospital fires, ICU fires are the most difficult to extinguish because of the need for transfer-dependent organ support therapy and invasive monitoring. The destruction of medical buildings, failure of medical equipment, and injuries to medical staff also likely delay care for the more critically ill.

There has yet been no systematic review on the causes, risk factors, and preventive measures of ICU fires. Many things delay the evacuation during a fire emergency, such as limited resources, miscoordination of the responders, or secondary incidents like explosions or blocked evacuation routes due to fire or smoke [1]. More importantly, research has found that at least 75% of fires are preventable [10]. Understanding previous experience with hospital and ICU fires is of great significance for future prevention efforts. Here we discuss the common causes and preventive measures of ICU fires with the aim of increasing medical staff's awareness of this danger, reducing the occurrence of ICU fires, or when they are unpreventable, encouraging them to respond quickly and properly protect the medical and patient population from major life-threatening injuries.

## METHODS

### Search strategy

We searched databases of PubMed, Cochrane, Embase, Web of Science, and Web of Baidu Academic from inception to 5 July 2024 for studies on ICU or hospital fires, using a combination of keywords related to intensive care units and fires (Text S1 in the **Online Supplementary Document****)**.

### Inclusion and exclusion criteria

We expected to find few studies on ICU fires in our search. Given the risk of injury and harm, we expected to find no randomised controlled trials; we included descriptive studies or case reports related to ICU fires or medical institution fires and injuries incurred from such incidents, regardless of the time of the study, place, and the language of publication. We excluded non-hospital fire studies and studies without available abstracts or full texts.

### Data collection and analysis

Two researchers (JFW, YYQ) screened the titles/abstracts, followed by the full texts of articles based on these pre-defined criteria, discussing any discrepancies in the process until a consensus was reached. The kappa statistic was 0.86, indicating a strong level of agreement [11]. They further screened the reference lists of the included articles for any potentially relevant studies. We then narratively synthesised the findings of the studies in our analysis.

### Quality or bias

We used the Joanna Briggs Institute (JBI) critical appraisal tools to evaluate the quality of the different kinds of studies. Specifically, the tools for case reports and cross-sectional studies consist of eight items each, with each item evaluated as ‘yes’, ‘no,’ ‘unclear’, or ‘not applicable’ [12,13].

 We classified the studies as high quality if the proportion of ‘yes’ was >90%, moderate quality if the proportion of ‘yes’ responses was between 75% and 90%, and low quality if the proportion of ‘yes’ responses was <75%.

## RESULTS

**Figure 1:**
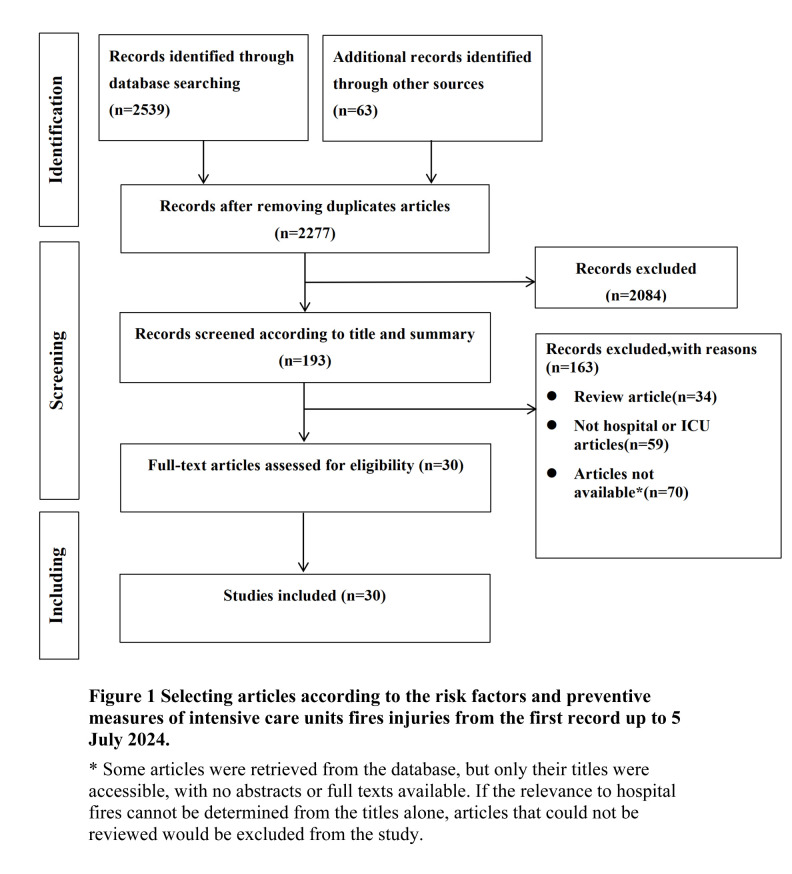
Flowchart of the study process. Some articles were retrieved from the database, but only their titles were accessible, with no abstracts or full texts available. If the relevance to hospital fires cannot be determined from the titles alone, articles that could not be reviewed would be excluded from the study.

The initial search retrieved 2602 studies, with 2277 remaining after deduplication, 193 after the title/abstract screening, and 30 after the full-text stage (**Figure 1**). Among them, 21 were case reports and nine were cross-sectional studies. They described 18 ICU fire cases, 11 operating room fires, and one outpatient area fire. There were 20 high-quality, seven moderate-quality, and three low-quality studies (**Table 1**, **Table 2**).

**Table 1:** Methodological quality of ICU fire case reports, per the JBI critical appraisal tool: case report and case series quality evaluation [12]

Study ID	Were the patient’s demographics clearly described?	Was the patient’s history clearly described and presented as a timeline?	Was the current clinical condition of the patient on presentation clearly described?	Were diagnostic tests or assessment methods and the results clearly described?	Was the intervention(s) or treatment procedure(s) clearly described?	Was the post-intervention clinical condition clearly described?	Were adverse events (harms) or unanticipated events identified and described?	Does the case report provide takeaway lessons?
Prasad et al., 2006 [14]	Yes	Yes	Yes	Yes	Yes	Yes	Yes	Yes
Du, 2007 [15]	Yes	Yes	Yes	Yes	Yes	Yes	Yes	Yes
Ashwani Gupta et al., 2009 [16]	Yes	No	No	Yes	Yes	No	Yes	Yes
A Comprehensive Media Report, 2011 [17]	Yes	Yes	Yes	Yes	Yes	Yes	Yes	Yes
Almeida et al., 2012 [18]	Yes	Yes	Yes	Yes	Yes	Yes	Yes	Yes
González et al., 2013 [19]	Yes	Yes	Yes	Yes	Yes	Yes	Yes	Yes
Gorphe et al., 2014 [20]	Yes	Yes	Yes	Yes	Yes	Yes	Yes	Yes
Kim et al., 2014 [21]	Yes	Yes	Yes	Yes	Yes	Yes	Yes	Yes
Rispoli et al., 2014 [22]	Yes	Yes	Yes	Yes	Yes	Yes	Yes	Yes
Kelly et al., 2014 [23]	No	No	No	Yes	Yes	No	Yes	Yes
Kelly et al., 2014 [24]	Yes	Yes	Yes	Yes	Yes	Yes	Yes	Yes
Tan et al., 2015 [25]	Yes	Yes	Yes	Yes	Yes	Yes	Yes	Yes
Lian et al., 2016 [26]	Yes	Yes	Yes	Yes	Yes	Yes	Yes	Yes
Bosack et al., 2016 [27]	Yes	Yes	Yes	Yes	Yes	Yes	Yes	Yes
Gupta et al., 2018 [28]	Yes	Yes	Yes	Yes	Yes	Yes	Yes	Yes
Kim et al., 2018 [29]	Yes	Yes	Yes	Yes	Yes	Yes	Yes	Yes
Adams et al., 2020 [30]	Yes	Yes	Yes	Yes	Yes	Yes	Yes	Yes
Greenidge et al., 2021 [31]	Yes	Yes	Yes	Yes	Yes	Yes	Yes	Yes
Choi et al., 2022 [32]	Yes	Yes	Yes	Yes	Yes	Yes	Yes	Yes
Paliwal et al., 2022 [33]	Yes	Yes	Yes	Yes	Yes	Yes	Yes	Yes
Aldridge, 2024 [34]	Yes	Yes	Yes	Yes	Yes	Yes	Yes	Yes

ICU – intensive care unit, JBI – Joanna briggs institute

**Table 2:** Methodological quality of ICU fire cross-sectional studies, per the JBI critical appraisal tool: prevalence study and analytical cross sectional study [13]

Study ID	Were the sources of research materials on hospital fires described in detail?	Were the causes, geographic scope, or timing of hospital fires described in detail?	Were the measurement methods for exposure factors reliable and valid?	Were there objective and consistent criteria for the outcomes of hospital fires?	Were confounding factors identified?	Were measures taken to control confounding factors?	Were the measurement methods for outcome indicators reliable and valid?	Were the data analysis methods appropriate?
Haith et al., 2012 [35]	Yes	Yes	Not applicable	Yes	No	No	Yes	Yes
Connor et al., 2018 [36]	Yes	Yes	Not applicable	Yes	Yes	Yes	Yes	Yes
Zhao, 2020 [37]	Yes	Yes	Not applicable	Yes	No	No	Yes	Yes
Zhao et al., 2021 [38]	Yes	Yes	Not applicable	Yes	No	No	Yes	Yes
Zhao et al., 2021 [39]	Yes	Yes	Not applicable	Yes	No	No	Yes	Yes
Zhao et al., 2021 [40]	Yes	Yes	Not applicable	Yes	No	No	Yes	Yes
Zhao et al., 2021 [41]	Yes	Yes	Not applicable	Yes	No	No	Yes	Yes
Malviya et al., 2022 [42]	No	Yes	Not applicable	Yes	No	No	Yes	Yes
Bayuo et al., 2023 [3]	Yes	Yes	Not applicable	Yes	No	No	Yes	Yes

ICU – intensive care unit, JBI – Joanna Briggs Institute

### The occurrence and injuries of ICU fires

**Figure 2:**
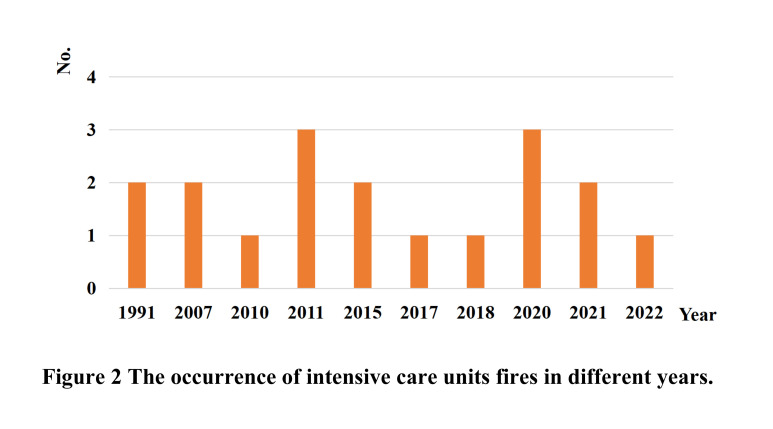
The occurrence of ICU fires across the years. ICU – intensive care unit

The ICU fires occurred in countries worldwide, with two cases in Canada, two in England, four in China, five in India, and one in Italy, Romania, Iraq, Ukraine, and the Republic of Senegal each (**Table 3**). ICU fires accounted for 50% in middle- and low-income countries, with heavy casualties. The earliest recorded ICU fire occurred in 1991 in a fragile neonatal ICU with no casualties [43]. The worst such event occurred on 13 July 2021, when a deadly fire tore through a southern Iraq hospital, killing 92 people and injuring 110 more. The incidence of fire outbreaks during the COVID-19 pandemic has increased approximately 3-fold compared with the last three years (**Figure 2**). The building destruction, economic losses, and public psychological trauma caused by ICU fires were not described in detail in the included studies.

**Table 3:** Characteristics of articles on ICU fire causes and prevention

Study, date or year of fire	Setting	Country	Cause/suspected cause	Deaths/injuries
Sankaran et al., [43], 4 August 1991	Royal University Hospital in Saskatoon (NICU)	Canada	Isolation socket pull	No injuries/deaths
Sankaran et al., [43], 8 August 1991	Hospital in Saskatoon (NICU)	Canada	Electrical fault	No injuries/deaths
Ashwani et al, [16], 12 September 2007	James Paget University Hospital	England	Faulty extraction ventilation system of the sluice room	No injuries/deaths
Rispoli et al., [22], 17 December 2010	Federico II University Hospital in Naples, Italy	Italy	A lightning strike caused the failure of an uninterruptible power supply located in the basement	No patients injuries/deaths. The smoke caused pharyngeal and conjunctival irritation in some staff members.
Kelly et al., [23,24], 22 November 2011	Royal United Hospital in Bath	India	Initiated inside the valve of the oxygen cylinder when transported	One case of burns to lower limbs, one smoke inhalation, one admitted for nebulisers
Paliwal et al., [33], 23 November 2011	Department of Anesthesiology and Critical Care at All India Institute of Medical Sciences in Jodhpur, Rajasthan, India	India	H-type oxygen cylinder caught fire while the cylinder valve was being opened	One technician suffered second-degree burns on the palm of one hand and third-degree burns on the other hand with thumb and palm involvement
Gupta et al., [28], 11 September 2015	Public sector tertiary care teaching hospital in Northern India	India	Exact cause required further investigations	No injuries/deaths
British Broadcasting Corporation, [44], 8 June 2017	Royal Stoke University Hospital	England	Started deliberately in a shared corridor between theatres and the ICU	No injuries/deaths
Zhao et al, [41], 2000–19	Several hospitals in China	China	Three due to electrical faults, fourth cause unknown	Four patients died
The Times of India, [45], 6 August 2020	Shrey Hospital	India	Spark from a short circuit in the fan or the board	Eight patients died, one paramedic staff hospitalised with 21% burns
The New Indian Express, [6], 8 September 2020	Government hospital in Vadodara in Gujarat	India	Short circuit in ventilator inside the ICU ward	No injuries/deaths
AlJazeera, [7], 15 November 2020	Piatra Neamt Hospital	Romania	An electrical short circuit	Ten patients died, six patients injured and in critical condition, one doctor had severe first- and second-degree burns covering 80% of his body
ReliefWeb, [8], 13 July 2021	The coronavirus ward at the al-Hussein Teaching Hospital located in Nasiriyah, southern Iraq	Iraq	An oxygen tank exploded	92 people died and 110 injured
Interfax-Ukraine, [46], 28 December 2021	Kosivska Central District Hospital	Ukraine	Memorial candle	Three patients died, three doctors received burns of varying severity
Le-Monde, [47], 25 May 2022	Hospital in the western Senegalese city of Tivaouane (NICU)	The Republic of Senegal	Electrical short circuit	11 babies died

ICU – intensive care unit, NICU – neonatal intensive care unit

### Causes of ICU fires

**Figure 3:**
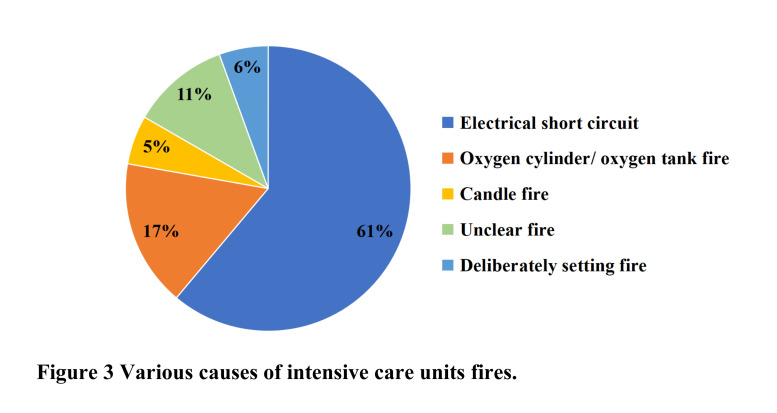
Percentages of causes of ICU fires. ICU – intensive care unit

The ICU fires were caused by electrical failure [6,7,16,22,41,43,45,47], oxygen cylinder-related fires [8,23,24,33], candle flames [46], arson [44,48], and fires of unknown cause [28,41] (**Figure 3**). The most common cause was electrical failure (61%); this included fires caused by unplugging sockets [43], power supply fires caused by lightning strikes [22], fan fires [45], air conditioning exhaust system fires [16], crane tower fires [41], ventilator fires [6], and circuit short circuit fires [7,47]. Elsewhere, large quantities of freeflowing oxygen were used to run ventilators and resuscitation bags [43]. The oxygen outlets leaked at the site where flow meters had been inserted [43]. In one more case, sparks caused by electrical faults quickly ignited and spread within equipment in an oxygen-rich environment [41].

The second most common cause (17%) of the fire was oxygen cylinder failure or explosion. ICU fires related to oxygen cylinders appeared to start inside the cylinder valve [23,24,33]. For example, there was one case of ICU fire triggered due to a spark from friction between the valve key and a residual antiseptic solution containing chlorhexidine gluconate I.P. 0.5% w/v and ethyl alcohol I.P. 70% v/v used for cleaning the cylinder and the hands of personnel and the fire was supported by large volumes of oxygen venting from the partly opened valve [33]. In other cases, one fire was caused by medical staff in a Ukrainian hospital ICU lighting candles in the ICU area in memory of a deceased COVID-19 patient [46]. There was one case in which a fire was deliberately set in a shared corridor between the theatre and the ICU at the Royal Stoke University Hospital [44,48]. The cause of the remaining two (11%) ICU fires was unknown [28,41].

### Lessons learned from ICU fires

ICU fire cases have highlighted two major issues: the prevalence of electrical fires and the extreme difficulty in safely transferring critically ill patients. In one case, the ICU was filled with thick, black, acrid smoke within ten seconds and visibility was reduced to less than a metre [24]. In another case, the ICU was filled with thick smoke after a minute and 38 seconds [45]. Elsewhere, visibility was nearly zero within three minutes [28]. In another outbreak, the windows of the ICU department were blocked and the window handles were (in places) not readily accessible [22]. During this emergency, it was discovered that nearly all department telephone lines were disabled; phone ringtones were almost inaudible because of the noise of the fire alarms therefore it was necessary to use mobile phones [22]. The transfer of critically ill patients often depends on oxygen, respiratory assistance equipment, and life-support medications. In two cases, cannulae and nasogastric tubes were inadvertently pulled out [23,24]; there, the evacuation of critically ill patients took about seven minutes [23,24]. During an ICU fire, critically ill patients were often transferred to other wards within the hospital, temporary ICUs, or nearby hospital wards [6,23,24,28]. Both patients and staff at the scene of the fire may experience post-traumatic stress disorder for months or even years afterwards [23,24].

## DISCUSSION

We found that, in many cases, staff, emergency teams, and other respondents were unprepared in cases of ICU fires [16]. It is therefore necessary to learn from these previous experiences experiences. Our review of 18 ICU fire incidents revealed that electrical failures and oxygen cylinder malfunctions were the primary causes of fires. Compared to other building fires, ICUs use many medical devices and have a high electrical load. The high-concentration oxygen environment in the ICU increases the likelihood of fire. The incidence of ICU fires has increased after the COVID-19 pandemic, particularly in low- and middle-income countries. Almost all ICU fires face the challenge of evacuating critically ill patients. The ideal strategy for ICU fire response is prevention before the fire occurs. We can thus stratify the following discussion about ICU fire risk factors into equipment-related issues, building infrastructure, human behaviour, and socioeconomic factors, emphasising preventive measures based on these factors in the process. This is especially pertinent because the number and severity of these events may be underestimated due to the absence of reporting regulations or formal critical incident reporting systems.

### Equipment-related factors

We found that electrical failures were the main cause of ICU fires. Previous studies have also identified electrical failures as the leading cause of hospital fires [49] or operating room fires [50]. Electrical faults remain the major cause of fires, consistent with findings from prior research [3,49–51]. ICU treatment depends on the continuous support via medical electrical equipment, including ventilators, vital sign monitors, ceiling pendant circuits, and continuous venovenous hemofiltration machines. Inappropriate placing of interior equipment or the use of unsafe devices significantly increases the risk of fires and related injuries. Factors that commonly trigger electrical short circuits and sparks include circuit overload, overheating, ageing equipment components, poor connections, loose joints, exposed wires, and socket overload. It is also worth nothing that there are no design-related safety codes specifying the location of oxygen outlets with respect to electrical sockets [43].

Most critically ill patients require continuous oxygen support, especially during transport. However, oxygen-related hospital fires have caused hundreds of deaths worldwide, particularly among critically ill patients during the COVID-19 pandemic [5,33]. As a strong oxidiser, oxygen constitutes about 21% of the atmosphere by volume, but fire risks increase significantly when its concentration exceeds 23%. Any small spark in a high-oxygen environment can quickly ignite a fire. Common causes of oxygen cylinder explosions include damaged valves, ageing cylinders, cylinder collisions, or leaking pipelines. Additionally, flammable and explosive materials in hospitals, such as instruments, chemical reagents, and bedding, can further intensify fires. Research has highlighted the urgent need for safety strategies to mitigate risks in flammable and explosive ICU environments [5].

Studies have reported a high incidence of fires in surgical rooms [52], with approximately 600 cases occurring annually in the US [20]. These fires were frequently associated with head and neck surgeries, where the use of electrocautery devices in oxygen-enriched environments after anaesthesia creates a high-risk situation [14,20,30,36,50,53,54]. From our experience, similar incidents can occur in ICUs during bedside tracheotomy or local surgical debridement. To minimise these risks, ICU physicians must exercise caution when using electrosurgical equipment and ensure oxygen concentrations are kept as low as possible, while maintaining adequate patient oxygenation.

### Building-related factors

The importance of emergency exits and escape routes in reducing casualties during fires has been widely recognised, especially for ICU patients who are frail and have limited mobility. Evacuating immobile patients is complex and often requires horizontal and vertical evacuation methods. We suggest that hospital design prioritises the needs of these vulnerable individuals to ensure their safety during a fire. A 2008 survey of ICU fire evacuation preparedness in 35 adult and paediatric ICUs in London found that fire evacuation preparedness was compromised by inadequate escape routes (62% had only one or two escape routes), a lack of portable monitoring equipment and emergency drug supplies, and no evacuation plan drills; only 20% of ICU ground floors accessible via means other than steps, such as ramps [55]. To improve safety, the design of new hospital buildings should include ways to prevent fires from spreading to ICUs [48], and should strive to place ICUs on lower floors [56]. If this is not feasible, other conditions that meet vertical evacuation requirements should be considered. Each department should ideally have three independent exits, with at least two supporting horizontal evacuations; step-free ground-floor access, either indoor or outdoor ramps, should be provided for vertical evacuation, while escape routes should be wide enough to accommodate hospital beds without adjustment [56].

### Human behavioural factors

Inappropriate use of fire within hospitals, such as smoking and discarding cigarette butts indiscriminately, lighting open flames, or even intentional arson, significantly increase the risk of accidental fires in the ICU. When an ICU fire occurs, the fire safety awareness of medical staff and patients plays a crucial role in determining the outcome. Strategies to minimise the harm of ICU fires include correctly using fire-fighting equipment, promptly sounding the alarm, being familiar with evacuation routes, and having department heads methodically direct the evacuation.

### Socioeconomic factors

The incidence of ICU fires is much higher in developing than in developed countries. Several studies have linked socioeconomic status, including low income, to a higher risk of fire-related injuries and deaths [49,57–59]. This may be related to outdated infrastructure, inadequate equipment maintenance, lack of financial resources to purchase safety equipment such as smoke detectors, and insufficient fire safety awareness [3].

### Fire prevention

Fire prevention measures recommended for ICUs specifically are similar to those suggested for other settings [28,48,56,60–65] (Table S1 in the **Online Supplementary Document**). The physical and chemical properties of medical electrical equipment should regularly be inspected, maintained, and recorded [5,48,56,60,65]. Large or specialised medical equipment should use independent power lines. Oxygen cylinders should be stored, handled, and used according to the supplier's instructions, following the correct operational procedures when administering oxygen, and always using oxygen cylinder bed supports [24,48,66,67]. Room oxygen monitors should be used where applicable, particularly in places where gas leakage remains an issue [68]. Fire alarms and smoke detectors should be installed in these areas to facilitate early detection of fire [5]. Regular checks should be conducted to ensure that fire escape routes and exits in all departments are truly unobstructed [28,48,56,60,61,65]. Strict inspections are necessary to verify that fire-fighting facilities are functioning properly [48,56,60,61,65]. Knowledge signs about safe evacuation should be prominently displayed on fire escape routes [48,56]. Fire extinguishers and fire-fighting equipment should be adequately provided and within their validity period to ensure they function properly when needed [48,56,64,65,69].

Although most ICUs reported departmental fire response plans, many lacked detailed guidance on practical aspects of patient evacuation [70]. A questionnaire-based assessment of ICU fire preparedness within a national public health care system in a high-income country found unexpectedly low fire preparedness levels in the ICUs, particularly in the lack of local-specific plans, staff education, and evacuation training for ICU evacuation in the event of a fire threat [70]. Even where national requirements mandate evacuation planning, education, training, and regular exercises, these are often only partially implemented [69].

A significant gap is the lack of practical evacuation exercises, as face-to-face training can conflict with health care workers’ patient care responsibilities. Several studies have suggested online fire safety training as a flexible and time-efficient alternative [48,71–75]. This approach complements in-person drills and health care workers who cannot attend regular training improve their fire safety and evacuation knowledge. Guidelines suggest conducting practical drill training or simulated evacuations every two years [48], with the sessions ideally covering the location and operation of manual fire alarm call points, oxygen shut-off valves, evacuation aids, evacuation routes and procedures, and the importance of keeping evacuation routes clear.

Prior research indicated that it takes an average of seven minutes to transfer each patient from an ICU bed to an ambulance [76]. Another prospective randomised controlled study simulated the impact of using a standardised checklist on evacuating patients under general anaesthesia from an operating room on the third floor of a medical centre, finding that the average time from the start of a simulated fire alarm to building exit was 593.8 seconds [77]. All fires start small, but can spread quickly. To address this urgent issue, smoke detectors and fire alarm systems should be installed to ensure timely alerts when a fire occurs [48,56,61,78,79]. Installing automatic sprinkler systems inwards can quickly extinguish or control a fire in its early stages, gaining crucial time for evacuation [48,56,63,78]. In daily operations, we posit that arranging bed assignments based on patients' conditions and mobility can enhance escape efficiency during a fire. The most critically ill and immobile patients should be positioned closest to the exits and evacuation routes, allowing health care workers to evacuate them quickly in an emergency. Patients with milder conditions and greater mobility can be placed further from the exits. Through reasonable bed arrangements and comprehensive emergency plans, the safety of patients and health care workers can be maximised during an ICU fire, improving escape efficiency and reducing fire-related harm and loss.

The typical doctors-to-bed ratio in ICUs is 1:1, and the nurse-to-bed ratio is usually 2.5–3:1. All health care workers, however, should know how to handle workplace fires. A cross-sectional study conducted among 202 health care workers in a tertiary care teaching hospital in the Marathwada region of Maharashtra between January and April 2016 found that 44.9% of respondents did not know how to use a fire extinguisher, and 72.28% were unaware of emergency telephone numbers in case of a fire outbreak [80]. All staff must know the locations of fire alarm call points, fire extinguishers, evacuation routes, and oxygen shut-off valves, especially in areas where patients are difficult to evacuate. Numerous studies have emphasised the role of fire safety training and fire simulation drills [3,16,25,31,71,75,81,82]. Fire training should ideally take place ‘in-situ,’ that is in the actual area of work [24]. Evacuation at night should also be practised to ensure that the ICU has enough appropriately trained staff on shifts to manage emergency evacuations at any time [48,83].

Each ICU bed should have appropriate and sufficient evacuation equipment stored in easily accessible locations. This includes portable oxygen cylinders and stands, evacuation aids (*i.e.* emergency stretchers, evacuation pads, or evacuation chairs), evacuation boxes (containing various equipment that might be needed during evacuation, including emergency medications, and first aid kits), and flashlights. These measures can significantly enhance the efficiency and safety of evacuations during emergencies such as fires [48]. If providing one emergency evacuation box per ICU bed is too resource-intensive, hospitals may consider sharing a box between nearby ICU beds. In hospitals without accessible ramps, emergency stretchers, evacuation pads, or evacuation chairs can help with vertical evacuation via stairs, but will require additional personnel. It is recommended that hospitals add convenient and accessible ramps to assist in evacuation during a fire [55]. However, it is important to consider that resources and conditions vary significantly between different ICUs. While some prevention and response measures may be feasible in resource-rich hospitals, they may be difficult to implement in resource-poor settings. For instance, it was reported that the Iraqi hospital where 92 people died lacked fire alarms or sprinkler systems [3]. The maintenance cost of evacuation equipment could also limit its availability in economically underdeveloped hospitals.

Artificial intelligence (AI) has made significantly advanced fire prevention technologies, including smoke detectors and automatic sprinkler systems. Recent research has focussed on optimising evacuation routes by combining physical models with computer-based numerical simulations [84–92]. This has led to the development of emergency evacuation route optimisation models, intelligent guidance systems, and fire warning systems [84–92]. Studies comparing hospital fire evacuations with and without these optimised systems showed that the optimised emergency evacuation route and intelligent guidance model effectively prevent crowd-induced congestion and chaos during evacuation [93]. Additionally, using virtual reality simulations of real fire scenarios, combined with AI-driven interactive training, can improve individuals' emergency response capabilities. As AI technology continues to develop, its applications in fire prevention and evacuation strategies are expected to expand, offering more effective solutions.

### Limitations

This study has several limitations. First, despite efforts to search various databases, it is possible that some ICU fire incidents were not thoroughly recorded or reported, leading to incomplete data and affecting the comprehensiveness of the analysis. Additionally, we had difficulties obtaining data from certain regions or countries, potentially inducing regional bias in the findings. Second, most studies on ICU fires rely on case reports with limited sample sizes and a lack of large-scale, systematic data support, which may have affected the generalisability of our conclusions. Third, with the continuous updating of technology and equipment, emerging fire risks may not have been fully identified and analysed.

## CONCLUSIONS

ICU fire incidents and their related mortality and morbidity are a significant public health concern, and preventing them is an urgent priority for hospitals. Adopting a prevention-first approach, combined with effective fire suppression measures, is essential. Key strategies include regular inspection and maintenance of medical equipment, strengthening fire risk assessments, the development and implementation of fire emergency plans, and enhanced staff training and awareness. These measures are crucial for improving survival rates during fire emergencies. In the future, more intelligent, mechanised, virtualised, and fully participatory integrated fire management models will help improve fire safety levels in hospital ICUs, ensuring the safety of patients and health care workers.

## Additional material


Online Supplementary Document
