# Supplementary material for: Causes and preventive measures for fire-related injuries in intensive care units: a systematic review
Source: J Glob Health. 2025 Feb 28;15:04043. doi: 10.7189/jogh.15.04043 (PMC11869519; doi:10.7189/jogh.15.04043)
Supplement: Online Supplementary Document [file jogh-15-04043-s001.pdf]

Supplement to: Wu JF, Yao YQ, Shang XL. Causes and preventive measures for fire-related injuries in intensive care units: a systematic review. J Glob Health. 2025;15:04043.

Text S1. Complete search strategy about ICU fires or hospital fires articles in databases of PubMed, Cochrane, Embase, Web of Science, and Web of Baidu Academic from the earliest record up to 5 July 2024.

I. PUBMED (Date Run: 05/07/2024)

"Hospital fire"[All Fields] OR "Hospital fires"[All Fields] OR "Hospital fire safety"[All Fields] OR ("intensive care units"[Title/Abstract] OR "intensive care unit"[Title/Abstract] OR "unit intensive care"[Title/Abstract] OR "icu intensive care units"[Title/Abstract] OR "burn units"[Title/Abstract] OR "coronary care units"[Title/Abstract] OR "intensive care units pediatric"[Title/Abstract] OR "intensive care units neonatal"[Title/Abstract] OR "recovery room"[Title/Abstract] OR "recovery room"[Title/Abstract] OR "ICU"[Title/Abstract]) AND ("Fires"[Title/Abstract] OR "Fire"[Title/Abstract] OR "fire extinguishing systems"[Title/Abstract] OR "open waste burning"[Title/Abstract] OR "spontaneous combustion"[Title/Abstract] OR "Wildfires"[Title/Abstract] OR "fire protection"[Title/Abstract] OR "fire safety"[Title/Abstract])

II. Web of Science (Date Run: 05/07/2024)

("Intensive Care Units" OR "Intensive Care Unit" OR "Unit, Intensive Care" OR "ICU Intensive Care Units" OR "Burn Units" OR "Coronary Care Units" OR "Intensive Care Units, Pediatric" OR "Intensive Care Units, Neonatal" OR "Recovery Room" OR "Respiratory Care Units") AND ("Fires" OR "Fire" OR "Fire Extinguishing Systems" OR "Open Waste Burning" OR "Spontaneous Combustion" OR "Wildfires" OR "Fire Protection" OR "Fire Protection" OR "Fire Safety") OR "Hospital fire" OR "Hospital fires" OR "Fire in ICU" OR "ICU fire"

III. Embase (Date Run: 05/07/2024)

("intensive care unit") AND ("fire" OR "fire protection" OR "fire safety") OR "intensive care unit fire" OR "hospital fire" OR "hospital fires" OR "fire in hospital" OR "hospital fire safety" OR "hospital fire risk" OR "intensive care unit fires" OR "fire in intensive care unit" OR "fire risk in icu" OR "fire in icu" OR "icu fire" OR "fire protection in hospital"

IV. Cochrane library (Date Run: 05/07/2024)

"Intensive care unit Fire"[ti,ab,kw] OR "Hospital fire"[ti,ab,kw] OR ("Intensive Care Units"[all trees] OR "Unit, Intensive Care)"[ti,ab,kw] OR "Intensive Care Unit"[ti,ab,kw] OR "ICU Intensive Care Units"[ti,ab,kw] OR "Burn Units"[ti,ab,kw] OR "Coronary Care Units"[ti,ab,kw] OR "Intensive Care Units, Pediatric"[ti,ab,kw] OR "Recovery Room"[ti,ab,kw] OR "Respiratory Care Units"[ti,ab,kw]) OR "Hospitals"[all trees] OR "Hospital"[ti,ab,kw]) AND ("Fires"[all trees] OR "Fire Extinguishing Systems"[ti,ab,kw] OR "Fire"[ti,ab,kw] OR "Open Waste Burning"[ti,ab,kw] OR "Spontaneous Combustion"[ti,ab,kw] OR "Wildfires"[ti,ab,kw] OR "Fire Protection"[ti,ab,kw] OR "Fire Safety"[ti,ab,kw]) OR "Hospital fires"[ti,ab,kw] OR "Fire in hospital"[ti,ab,kw] OR "Hospital fire safety"[ti,ab,kw] OR "Hospital fire risk"[ti,ab,kw] OR "Intensive care unit fires"[ti,ab,kw] OR "Fire in Intensive care unit"[ti,ab,kw] OR "Fire in Intensive care unit"[ti,ab,kw] OR "Fire in lCU"[ti,ab,kw] OR "Fire risk in lCU"[ti,ab,kw] OR "ICU fire"[ti,ab,kw] OR "Fire Protection in hospital"[ti,ab,kw]

V. Web of Baidu Academic (Date Run: 05/07/2024)

"Hospital fire" OR "Hospital fires" OR "hospital fire" OR "hospital fires" OR "Hospital fire safety" OR "hospital fire safety" OR "ICU fire" OR "ICU fires" OR "ICU fire safety" OR "intensive care units fire" OR "intensive care units fires" OR "intensive care units fire safety"

Table S1. Summary of Hospital Fire Prevention and Evacuation Guide published by the Pan American Health Organization in 2014 [1]

**Section**	**Content**
**Prevention**	● Materials used in the design and construction of hospitals must be noncombustible/nonflammable, must have adequate fire resistance ratings, and should not emit toxic gases/smoke during a fire.<br>● ICUs and emergency units should be located on the ground floor or first-floor level with dedicated access ramps.<br>● Each stairwell needs to have fire doors at each landing. There must be a minimum of two independent egress routes and exits for every location on every floor. The width of the corridor leading to the emergency exits (unobstructed) should be at least 2.4 m (7.9 feet). This will permit the transportation of hospital beds, mattresses, and so forth in the evacuation of non-ambulatory patients. Doors should be of the minimum width necessary to accommodate a stretcher (typically 1.25 m).<br>● Evacuation maps should be posted at the hospital's main access points to clearly identify egress routes. Egress routes and exits should be clearly identified.
**Suppression**	● A variety of smoke and heat sensors can be installed as part of a fire alarm system to detect fires that begin in low-traffic areas away from personnel/staff.<br>● Once a fire has been detected, a suppression system to extinguish the fire is required to minimize damage and avoid evacuation.<br>● A variety of firefighting equipment (such as fire extinguishers, water sprinkler systems, mist sprinkler systems, water hose reels, smoke extractors) can be installed in different locations in the hospital to combat specific types of fires.<br>● One of the most critical aspects of an effective suppression system for combating fires is planned preventative maintenance. Regular checks should be performed and documented. The National Fire Protection Association10 (USA) recommends that smoke detectors be replaced every 10 years.

**Evacuation**	● Once the fire alarm is triggered, there need to be designated personnel to investigate the reason for the alarm (and the possibility of a false alarm). They must also determine whether the fire is a small one that can be suppressed or whether evacuation is necessary.<br>● In the case of a fire, evacuation is performed once the preventative and suppression measures described earlier have failed to contain the fire and lives are under immediate threat. It is important to note that there is no fixed methodology for evacuations; the procedure will vary for each individual health care facility.<br>● Every shift should have health and safety officers or wardens on-site who are trained and knowledgeable regarding fire response and evacuation procedures. All hospital staff should have working knowledge of the evacuation routes and which one to take, based on the type of evacuation (horizontal, vertical, or shelter in place) and as instructed by the hospital's incident commander.<br>● If you hear the fire alarm or see flashing lights, close all fire doors in your area. Ensure that egress corridors are clear to allow movement of patients and equipment. Locate and secure patients' medical records and medical supplies. Ready evacuation transport equipment such as wheelchairs, blankets, and gurneys. Move people to designated assembly points.<br>● Patients may require specific life support equipment (e.g., ventilators) that should accompany them when they evacuate. Equipment that is battery operated should be regularly checked as past of the hospital's programmed maintenance.Specific medications that patients need for treatment should also accompany them when they evacuate.
**Evacuation Training Drills**	● Activation. Upon the sound of the fire alarm, it is expected that hospital staff will activate a practiced system or sequence of activities in response.<br>● Training of Staff. General training of all staff should include, but not be limited to, the following: Training on how to lift and move patients. Training on how to use fire extinguishers. Training on what to do if they see a fire. Training on what

	to do if they hear the alarm and see the flashing lights. Specific training defines the roles and responsibilities of each staff member.<br>● Action cards are brief summaries that define each role in an emergency situation and detail the tasks assigned to this role.<br>● All training simulations and fire drills need to be scheduled and performed regularly. Regular training and simulations are necessary to improve the response and safety of the staff and patients. Through regular training drills and simulations, they should know the details of the evacuation plan and be aware of their roles and responsibilities.

REFERENCES

1 Pan American Health Organization. Hospitals don't burn! Hospital Fire Prevention and Evacuation Guide. Washington DC, US: Pan American Health Organization; 2014. Available: https://iris.paho.org/handle/10665.2/34976. Accessed: 16 January 2025.
